# Effect of the Cooling Liquid on the Milled Interface in the Combined Process of Milling and Direct Metal Deposition

**DOI:** 10.3390/ma17133119

**Published:** 2024-06-25

**Authors:** Sergei Egorov, Timo Schudeleit, Konrad Wegener

**Affiliations:** 1 Inspire AG, Technoparkstrasse 1, 8005 Zurich, Switzerland; timo.schudeleit@inspire.ch (T.S.); wegener@iwf.mavt.ethz.ch (K.W.); 2 Institute for Machine Tools and Manufacturing (ETH Zürich), Leonhardstrasse 21, 8092 Zurich, Switzerland

**Keywords:** additive manufacturing, directed metal deposition, hybrid process, cooling liquid, Inconel 718

## Abstract

The combination of Direct Metal Deposition (DMD) with milling offers numerous advantages for the manufacturing of complex geometry parts demanding high dimensional accuracy and surface quality. To reach this, a process strategy alternation between both processes is often required, leaving the milled surface with a layer of cooling fluid before adding material by DMD. This paper investigates the effect of cooling liquid on the milled interface in the combined process of milling and DMD. Five different interface conditions were examined, employing four distinct cleaning techniques to assess their impact on the quality of the interface. Key metrics analysed included hydrogen content, carbon content, and porosity levels at the interface. Cleaning techniques were evaluated to determine their necessity in enhancing the interface quality in the combined DMD and milling production process. Results from this study provide essential insights into the optimal cleaning requirements for improving the interface integrity in hybrid manufacturing processes, which could lead to more reliable and efficient production methods in industrial applications.

## 1. Introduction

Direct Metal Deposition (DMD) is a metal AM technology that involves the layer-by-layer deposition of metal powder using a focused energy source (e.g., a laser) to melt and fuse the powder particles onto a substrate or previously deposited layer [1]. This process allows for repairs, applying wear- and corrosion-resistant coatings, and creating complex metal parts. This innovative method can be combined with conventional subtractive manufacturing techniques, such as milling, to form a hybrid manufacturing process that increases the dimensional accuracy and surface finish of the resulting parts. Using the advantages of both additive and subtractive methods can significantly increase manufacturing flexibility, reduce material waste, and enable the production of components with complex geometries that were previously almost impossible to fabricate by conventional manufacturing methods. It opens a new perspective in manufacturing technology, offering a way to more efficient, sustainable, and customisable production of highly loaded products for various applications from medical to aerospace.

Aerospace parts are often made of hard-to-machine alloys (e.g., nickel- or titanium-based alloys), which represents a technological challenge in machining from an economic and technological point of view. One of the most commonly used nickel-based superalloys is precipitation-hardening nickel-based alloy Inconel 718 (IN718), developed by the Nickel Corporation in the 1950s. It has a high level of mechanical properties, including superior creep resistance, excellent oxidation and corrosion resistance at elevated temperatures up to 650 °C, and good weldability [2,3]. These properties ensure its wide application in the aerospace industry.

Nevertheless, Inconel is a hard-to-machine material [4], and the heat generated by machining is excessive due to its high toughness and strength, even at high temperatures. Due to its low thermal conductivity, heat is concentrated on the tool surface during the machining of Inconel, which leads to increased tool wear. During the machining of these alloys, the temperature and stresses near the cutting tool edge are significantly high, making it susceptible to damage such as flank wear, notching wear, and edge chipping. These issues result in a reduced tool lifespan and a decline in the integrity of the finished surface [5]. Additionally, welding and adhesion of the machined material to the cutting tool frequently occur, causing severe notching and altering the tool’s rake face due to the subsequent pull-out of the coating and tool substrate [6]. Therefore, various cooling and lubrication methods are used in the milling process to reduce cutting forces and tool wear. For nickel alloys, a great variety of methods such as dry cutting, flood cutting, cryogenic cutting, and cryogenic minimum quantity lubrication can be used for this purpose [7]. Despite the variety of methods, cutting flood cutting using water-oil emulsions with a concentration of about 3–9% or minimal quantity lubrication (MQL) is most common in industry [8]. Based on this, it was concluded that the use of cutting fluids in both the combined DMD and milling process will be unavoidable for hard-to-machine materials in most cases. It may lead to interface contamination in the sequential surfacing and milling process. The following sections present research on the combined process, highlighting the significance of ongoing investigations in this field and reviewing the current state of the art.

Researchers [9,10,11,12,13] studied the machinability of IN718 produced by AM methods. Their findings showed the influence of the porosity and heat treatment on the cutting forces, tool wear, or surface roughness. These results indicate a strong interest in the topic of machining parts produced by AM methods.

Song et al. [14] reported on a hybrid approach called “3D welding and milling” that combines gas metal arc welding with milling. The study focuses on the early stages of process development and characterises parts fabricated using this approach. It delves into the results, highlighting the unique capabilities of this hybrid process in fabricating metallic prototypes and potentially overcoming limitations of surface finish and accuracy often associated with additively manufactured parts.

Soffel et al. [15] focused on the fabrication of parts using casting, interface milling, and DMD. They reveal that different processing routes impact the mechanical properties and bonding quality of the final product. This highlights the importance of understanding how DMD and milling interact at the interface level.

Authors like Barckhoff [16] and Alsher et al. [17] have noted that before laser welding, any lubricants on the surface should be cleaned off to prevent the formation of gas bubbles and pores. Cortina et al. [18] analysed the impact of using cutting fluid in a hybrid manufacturing process that combines additive and machining operations of IN718. The paper examines whether DMD after machining is feasible without requiring intermediate cleaning to remove residual cutting fluids. It concludes that if the part surface is not adequately cleaned after machining, it could lead to poor quality in the subsequent laser additive operation. However, in this work, only the porosity of the prepared samples was evaluated. In addition, the samples themselves are only single-layered on a forged substrate, which does not fully reflect the real combined manufacturing process of 3D parts, which is a multilayer cladding on an already deposited part of the workpiece.

It is known that cutting fluids, remaining in the zone of interaction with the laser during further cladding, are subjected to heating, vaporisation, and dissociation, which can accelerate the diffusion of hydrogen or carbon into the material, leading to undesirable effects such as hydrogen embrittlement. Aiello et al. [19] investigated the effect of H content in IN718 manufactured by Selective Laser Melting (SLM). Tests were carried out at hydrogen concentrations up to 28 parts per million (ppm). The results show hydrogen embrittlement at hydrogen concentrations starting from 2.7 ppm (drop in elongation at break by about 10%). This suggests a significant effect of hydrogen concentration on the properties of IN718.

Thus, the problem of using coolant in hybrid processes is an important issue that requires further study. Research in this direction can assist in determining the need for an additional technological cleaning operation, which is likely to increase the cost of the process and the final product. The aim of this study is to investigate the effect of interface cleaning methods in a combined DMD + milling process on the quality of the interface itself by studying the porosity, carbon and hydrogen content on multi-layer samples. To the best of the authors’ knowledge, the impact of liquids on the subsequential DMD process has not been investigated in such detail previously.

## 2. Materials and Methods

### 2.1. Experimental Setup

The nickel-based superalloy Inconel 718 (Oerlikon Metco AG, Oerlikon, Switzerland) was selected for the experiments. A 5-axis combined milling and DMD machine GF HPM 450 U (Georg Fischer AG, Schaffhausen, Switzerland) was used for additive buildup of specimens. Additive manufacturing of the DMD structures was performed with the integrated laser processing system Ambit S5 from HMT (Hybrid Manufacturing Technologies, Midlands, UK) that includes an IPG YLR-1000-MM-WC (IPG Photonics Corporation, Oxford, MA, USA) fibre laser. The working distance of the processing head was set at 8.0 mm, which resulted in an approximate melt pool width of 2.2 mm. Argon is used as shielding gas, nozzle protection, and powder carrier gas at flow rates of 8 L/min, 6 L/min, and 4 L/min, respectively. The material composition of the used powder is detailed in Table 1.

The milling step was carried out with a metalworking fluid (MWF) semi-synthetic water-miscible cooling lubricant Viscotex Zubora 67H Ultra (VISCOTEX Schmiertechnik AG, Altstätten, Switzerland). The selected MWF was recommended for the milling of IN718 by the machine manufacturer. The density of the MWF is 0.97 g/cm^3^ at 15 °C. The tests were conducted with 100% lubricant and 5% concentration emulsion.

For the milling tests, a Fraisa W00410404 end mill with W50410013 cutting inserts (Fraisa SA, Bellach, Switzerland) was used. Cutting parameters were chosen in accordance with recommendations from the manufacturer: cutting speed—40 m/min, feed per tooth—0.04 mm, axial infeed depth—1 mm, radial infeed depth—15 mm (30 mm is maximal for this tool), spindle speed—320 revolutions per minute, feed rate—51 mm/min.

### 2.2. Fabrication of the Specimens

**Figure 1:**
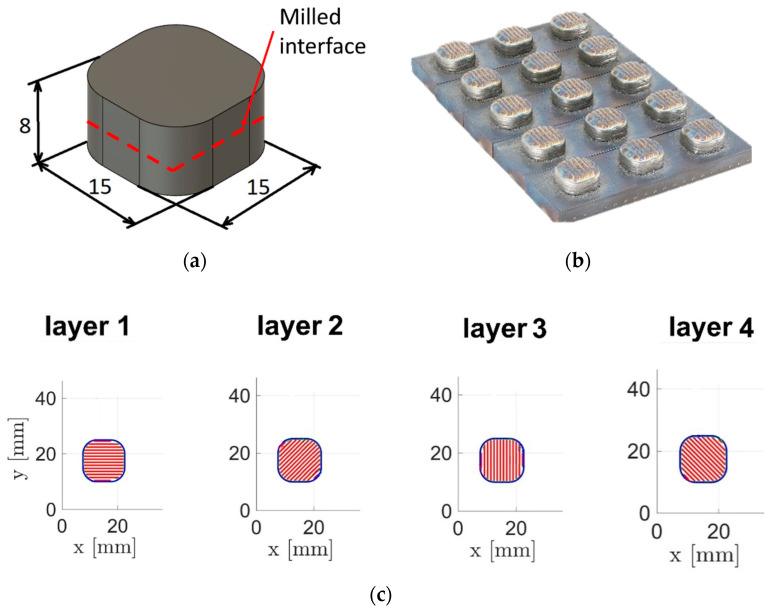
(**a**) Specimen geometry, (**b**) photo of the specimens, and (**c**) raster orientation.

The geometry of the specimens is shown in Figure 1. The scanning raster orientation was changed by 45° for each layer (Figure 1b). The scanning speed was 200 mm/min for the contour and 333 mm/min for the raster [15]. The specimens were produced segment by segment, with intermediate machining between the segments. The segment height is 4 mm, and the whole specimen is 8 mm. These dimensions were chosen to be representative of process conditions in a massive segment. Five sets of three specimens were fabricated for each lubricant concentration with different cleaning techniques applied to the interface between segments (Table 2). Method P1 was chosen as the reference, demonstrating the cladding process on top of the uncleaned and unprocessed DMD part. Method P2 includes manual cleaning with ethanol—a proven, but manual technique. Method P3 includes cleaning with air blasting—a method which can be relatively easily implemented and automatised but might require machine modification. Method P4 includes the laser treatment of the surface with a defocused laser beam with a power of 1000W to evaporate liquid before cladding. Defocusing was carried out by moving the nozzle 10 mm in the +Z direction. This method can be easily implemented by slight modification of G-code and requires no modification of the current setup. Method P5 includes no specific cleaning besides tilting the machine table by 90° for 5 s to let the liquid drop off from the milled surface. This method is also easily implementable and the fastest, but the cleaning quality is reduced to a very low level.

This lubricant was applied to the corresponding specimens according to Table 2. Two sets of tests were carried out—with a concentration of 5% of the lubricant in the cutting liquid for the first set and 100% for the second one. These concentrations were chosen as the most commonly used for the machining of IN718. The specimens are named according to the following rule—each sample is named PS_C, where S (from 1 to 5) indicates the process chain and cleaning method according to Table 2, and C is the concentration of the lubricant in the cutting liquid (5 or 100). For example, P5_100 means that 100% concentration of the lubricant in the cutting liquid and the cleaning method P5 were used.

### 2.3. Experimental Characterisation

The cross sections were ground flat with SiC papers from 240 to 4000 and polished with 1 μm and 0.1 μm diamond suspension. The cross sections were analysed on a Keyence VHX-1000 digital microscope (Keyence, Osaka, Japan). Kalling II was used as an etchant to reveal the microstructure. In the polished condition, the porosity was determined from ten cross-sections by image analysis with the software ImageJ (version 1.51p, ImageJ, Bethesda, MD, USA). 

A Zeiss EVO 10 scanning electron microscope (SEM, Carl Zeiss AG, Jena, Germany) equipped with an Oxford Instruments X-MaxN detector for energy-dispersive X-ray spectroscopy (EDS, Oxford Instruments, Abingdon, UK) was used for SEM and EDS analysis of the specimens. The EDS method can be used for qualitative analyses of light elements (B, C, etc.), although even quantitative analyses can be performed with a proper selection of parameters [20].

### 2.4. Hydrogen Measurement

Hydrogen content determination was conducted using the hot gas extraction (HGE) method on the LECO ONH 836 (LECO, Ann Arbor, MI, USA) machine. At least three specimens (~0.5 g mass of each specimen) were extracted and measured for each DMD cube for hydrogen determination. The specimens were extracted from the middle of the specimen from the interface zone. 

To calculate the hydrogen concentration change in the specimen over time, Fick’s diffusion law calculations were performed. Fick’s Laws of Diffusion provide the foundational principles for describing the transport of atoms within a medium. Fick’s First Law states that the flux of atoms moves from regions of high concentration to low concentration, proportional to the concentration gradient. Mathematically, it is expressed as:

(1) $$J=-D\frac{\partial D}{\partial x}\;,$$

where *J* is the diffusion flux, *D* is the diffusion coefficient, *C* is the concentration, and *x* is the spatial coordinate.

Fick’s Second Law, which is derived from the first law under the assumption of a constant diffusion coefficient, addresses the change in concentration over time:

(2) $$\frac{\partial C}{\partial t}=D\frac{\partial^{2}C}{\partial x^{2}}$$

This equation describes how diffusion causes the hydrogen concentration to evolve within the Inconel 718 specimen.

The diffusion process was modelled using a Gaussian distribution, centred at the middle of the specimen with an offset of 0.4 mm, to represent the hydrogen concentration profile. This assumption represents the increased H concentration in the layer below the interface. The Gaussian model was chosen for its physical relevance in many diffusive processes.

The concentration profile at any position *x* and time *t* was estimated using the equation:

(3) $$C\left(x,t\right)=C_{m,init}exp\left(-\frac{{\left(x\right)}^{2}}{2\sigma_{0}^{2}}\right)$$

where *C_m,init_* is the peak concentration at the initial time.

The peak concentration was adjusted based on the spread of the distribution to ensure mass conservation:

(4) $$C_{m,init}=C_{m,current}\times \frac{\sigma_{current}}{\sigma_{0}}$$

where *σ_current_* is the standard deviation after the selected time, calculated as $\sigma \left(t\right)=\sqrt{4Dt}$.

## 3. Results

**Figure 2:**
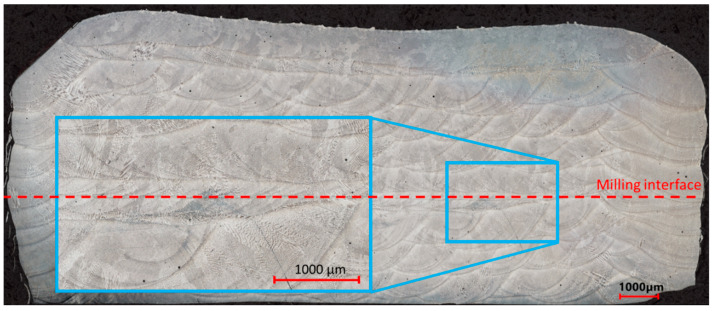
Etched cross-section of the specimen P2_5.

An overview of the specimen P2_5 cross-sections is shown in Figure 2. In the figure, the dashed line indicates the interface between the lower and upper sections of the specimen. It can be seen that no additional porosity is observed at the milled interface, except for a minimal number of gas-entrapped pores that occur throughout the specimen due to the non-ideality of the powder material.

**Figure 3:**
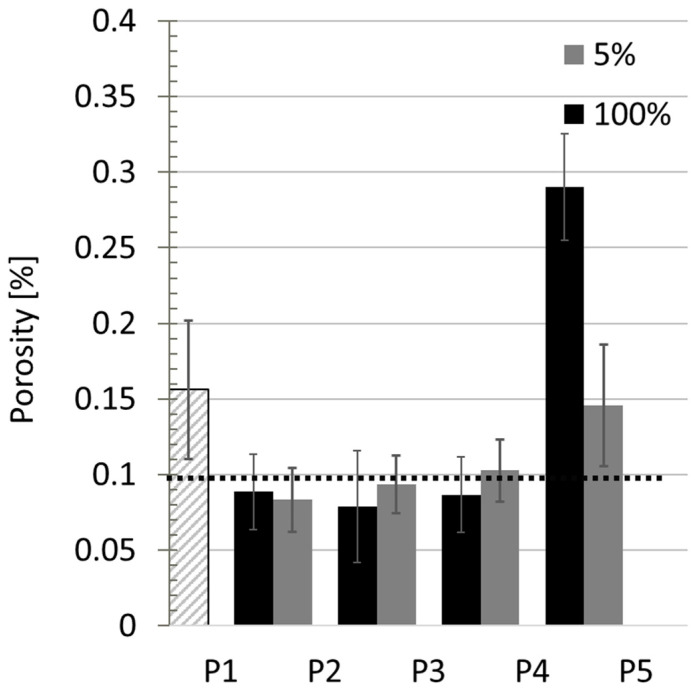
Porosity levels for the obtained specimens from the five different process chains according to Table 2 and two different concentrations of the lubricant. Dotted line shows the target value—DMD-fabricated specimen with no interim process interruption. P1 is marked with a different pattern since it does not refer to any cleaning technique and liquid concentration.

The results of porosity measurement by the optical method are presented in Figure 3. The porosity was measured on the polished specimens in three areas of the interface for each specimen. The measurements took place no further than one layer away from the interface. The porosity result for a DMD specimen, fabricated without interim milling, is 0.1%. The results of specimen P1_5 show porosity values comparable to P5_5. The porosity level of the specimen P2_5 was chosen as a base level due to the most careful cleaning being applied. The specimen P1 shows higher porosity values in comparison with P2, P3, and P4 due to the non-smooth and oxidized surface generated as the last layer by DMD in P1 compared to the milled surfaces in the other process chains. The results for the three applied cleaning techniques (P2, P3, P4) show very close results at approximately 0.1%, not depending on the cutting liquid concentration. The specimen P5_100 shows the highest porosity for these tests, approximately 0.3%. 

**Figure 4:**
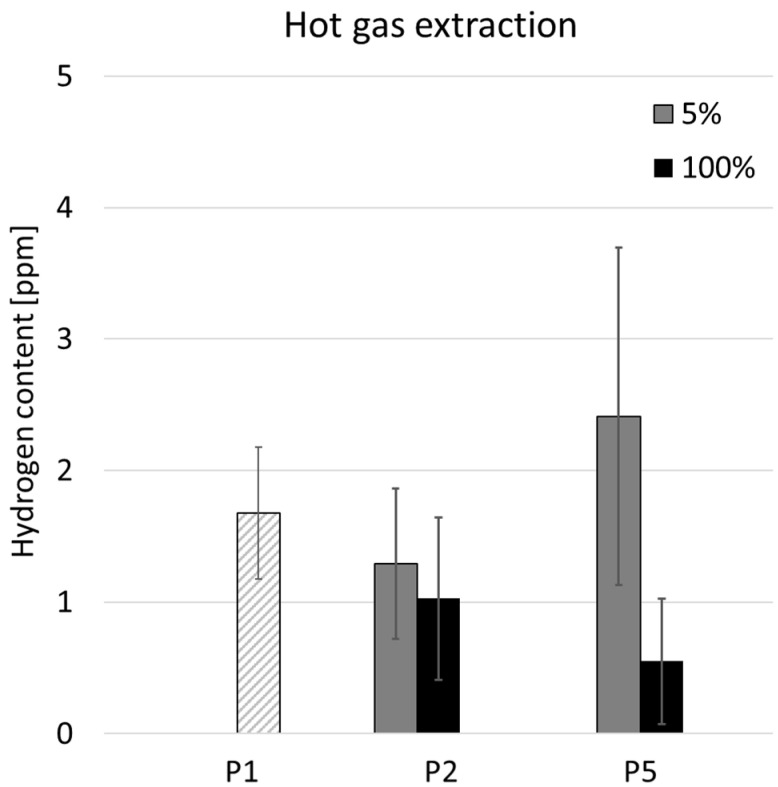
Hydrogen content of the investigated specimens, measured by the hot gas extraction method. P1 is marked with a different pattern since it does not refer to any cleaning technique and liquid concentration.

The hot gas extraction method was used to measure the hydrogen content, and the most representative specimens P1, P2, and P5 were selected for analysis. The measurement results are shown in Figure 4. The graph shows that the hydrogen content in the interface of samples P1, P2, and P5_100 is below 2 ppm. Sample P5_5, on the other hand, shows an average value slightly higher than 2 ppm. 

**Figure 5:**
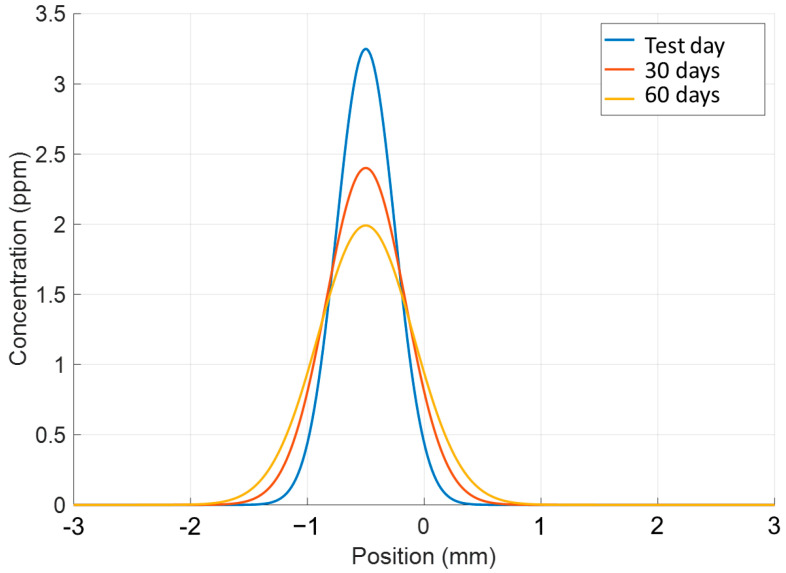
Evolution of hydrogen concentration in IN718 (P5_5).

The hydrogen concentration in the specimens was measured 60 days after they were fabricated. They were stored at 20 °C and cut right before the HGE. Therefore, it was assumed that the concentration in the specimen interface decreased due to diffusion. To calculate the change in hydrogen concentration in the specimen with time, Fick’s diffusion law calculations were carried out. The diffusion coefficient D was assumed to be 5 × 10^−15^ m^2^/s, according to Aiello et al. [19]. The average value for the P5_5 sample, which is 2 ppm, was used in the calculations. The equations used were described in Section 2, and the results are shown in Figure 5.

It can be seen that the width of the distribution increased over time, and the peak concentration decreased. The calculation showed that immediately after fabrication the concentration was 3.2 ppm. This change is relatively small for such a time interval due to the low Inconel diffusion coefficient and the absence of elevated temperature.

Also, by extracting the HGE sample from the middle of the DMD sample, the capture of a small part of the material not exposed to hydrogen can be assumed, which might have affected the measurement results to a small extent, reducing them. Therefore, the authors estimate that the real concentrations in the interface zone are slightly higher.

**Figure 6:**
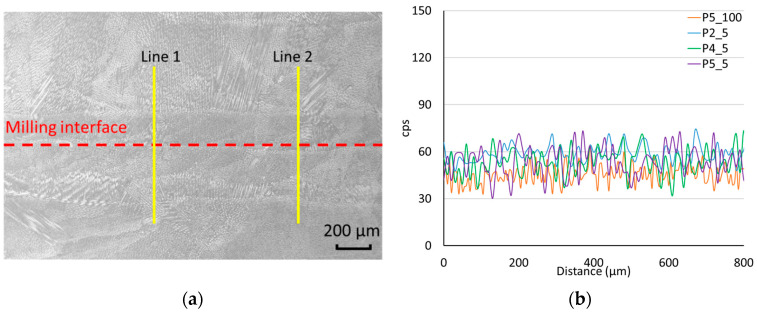
Carbon content determination. (**a**) SE image of the interface P2_5. (**b**) Comparison of representative linear measurements for the listed specimens.

The results of carbon content measurement by EDS are shown in Figure 6. Four lines were measured in the interface area for each specimen. A secondary electron (SE) image with the marked milling interface and measured carbon content lines is shown in Figure 6a. Examples of the obtained linear element scans are shown in Figure 6b. None of the measured specimens show any detectable increase in carbon content in the observed interface.

## 4. Discussion

An important parameter in this process is the temperature after cladding, before milling, and after milling, which can affect the porosity and microstructure of the parts. The specimen surface temperature after cladding was similar for all specimens due to the same process parameters and, therefore, was not considered. After cladding, there was a dwell time applied for about 15 min for technological reasons (tool change, part height measurement, etc.), when the specimens cooled down to temperatures below 50 °C. Also, a coolant was used during the milling process which ensured that the temperature was reduced without further affecting the material structure. This also ensured that the temperature of the specimen surface after milling and before the start of the cladding of the next segment was in the ambient temperature range.

Regarding the laser cleaning method, it may be noted that laser surface treatment involves high-temperature thermal processing, which can critically impact the final product quality. High temperatures can lead to thermal erosion and surface imperfections, such as micro-cracks and cavities, limiting the practical applications of laser processing [21]. However, in this work, the laser parameters were chosen to minimize the thermal effects but, at the same time, effectively remove the liquid from the investigated area. No remelted layer or defects due to laser influence are observed on cross-sections of P4 specimens.

It is important to control the porosity level since it has a negative effect on the mechanical properties [22,23]. According to the porosity analysis, it can be concluded that the cutting liquid has a negative influence on the quality of the interface in the segmental cladding. The reference specimen P1 shows higher porosity values, probably due to the fact that the surface is rough (typical for the DMD process). The air or laser-assisted cleaning methods showed almost the same effectiveness in reducing porosity. The specimen with ethanol cleaning showed the lowest porosity value. The specimen P5 shows the highest porosity values. Porosity is probably formed due to the vaporising liquid bubbles trapped in the molten material. Therefore, the different cleaning methods showed comparable effectiveness as they removed cutting liquid from the surface. 

Analysis of carbon content did not detect increased values at the interface in any of the samples studied. The assumption of carbon saturation of the alloy is related to the composition of the lubricant—consisting of more than 80% carbon. These results may be due to the fact that the lubricant present in the cutting fluid evaporates in the process faster than the diffusion of carbon into the alloy.

For high-performance materials like Inconel 718, manufacturers strive to reduce hydrogen levels to below 1 ppm if possible, especially for critical applications [19]. Control of the hydrogen level is important due to the risk of hydrogen embrittlement [24,25]. The hydrogen content analysis showed that the hydrogen concentration in the specimens is slightly elevated for this material. Ideally, keeping the hydrogen content as low as possible minimises the risk of hydrogen embrittlement. Sample P5_5 showed an increased hydrogen content of up to 3 ppm, which, in accordance with the data from the research [10], can lead to a decrease in fracture strain from 40% to 30% being attributed to hydrogen embrittlement. The elevated hydrogen content in specimens produced with 5% lubricant at the interface could be due to the vaporization and dissociation of water and oil in the plasma created by the laser beam. Hydrogen is then resolved in the metal melt pool.

These defects can seriously affect the strength and durability of the material, especially in critical applications where high reliability is required. The importance of controlling the hydrogen content cannot be overestimated, as even a slight increase in the content of this element leads to a significant reduction in the ductility of the alloy through the mechanism of hydrogen embrittlement. The results of the study emphasise the need for the implementation of cleaning methods prior to cladding in the studied process to reduce porosity and hydrogen uptake.

## 5. Conclusions

The results obtained allow a comparison of segment-by-segment cladding without and with the use of milling between segments, as well as different methods of cleaning the obtained interface. The following conclusions can be drawn:

- It is important to perform the top milling of the “base” segment in a segment-by-segment DMD process as it flattens the surface, resulting in a higher interface quality;
- In terms of porosity, tilting of the machine table has been shown to be the least effective compared to all the other applied methods. Air blasting and laser-assisted cleaning methods have proven effective in reducing porosity and can be relatively easily implemented in the existing process chain as it can be automated. Ethanol cleaning has shown the best results in this respect but is the most economically unfavourable solution due to the difficulties in automation and implementation in a continuous process;
- Cleaning the part surface is necessary not only to reduce porosity but also to reduce hydrogen saturation on the surface, which can lead to hydrogen embrittlement. This effect is more pronounced with an emulsion than with a pure lubricant because of the significant influence of water in this liquid.

Thus, automated cleaning methods, such as laser or air blasting, are generally sufficient and help avoid the constraints of dry cutting, which is particularly disadvantageous for hard-to-machine materials like nickel-based superalloys. However, for critical parts, manual cleaning with ethanol is advised to enhance interface quality, despite a reduction in productivity. This approach ensures the reliability needed for such components, while less critical applications may benefit from the efficiency of automated techniques.

## Figures and Tables

**Table 1:** Inconel 718 composition.

Element	Fe	Ni	Cr	Nb + Ta	Mo	Ti	Al	C	N	O
wt.%	18.1	53.4	18.9	5.1	3.05	0.88	0.44	0.05	0.02	0.01

**Table 2:** Description of the process chain and intermediate cleaning.

Interface Treatment	Fabrication Details			
	1st Segment	Milling	Cleaning	2nd Segment
P1	Cladding	-	-	Cladding
P2		Milling	Ethanol cleaning	
P3			Air Blasting	
P4			Laser Cleaning	
P5			Table Tilting	

## Data Availability

The datasets generated during and/or analysed during the current study are available from the corresponding author on reasonable request.
